# Genotyping of Clinical Samples of Methicillin-Resistant *Staphylococcus aureus* Isolates in Isfahan Using Multilocus Sequence Typing (MLST)

**DOI:** 10.1155/cjid/7307394

**Published:** 2025-06-01

**Authors:** Yasaman Ahmadi, Farnoosh Shekarchizadeh, Farnood Khajavirad, Afrouz Shekarchizadeh, Dariush Shokri

**Affiliations:** ^1^Department of Microbiology, Kish International Branch, Islamic Azad University, Kish Island, Iran; ^2^Department of Genetics, Faculty of Science, Shahrekord University, Shahrekord, Iran; ^3^Department of Biology, ShK.C., Islamic Azad University, Shahrekord, Iran; ^4^Biotechnology Research Center, ShK.C., Islamic Azad University, Shahrekord, Iran; ^5^Nosocomial Infection Research Center, Isfahan University of Medical Sciences, Isfahan, Iran

**Keywords:** antibiotic resistance, methicillin-resistant-*Staphylococcus aureus*, multilocus sequence typing, *Staphylococcus aureus*

## Abstract

Effective management of hospital-acquired infections caused by *Staphylococcus aureus* necessitates a comprehensive understanding of bacterial characteristics. The genotyping of clinical samples of methicillin-resistant *S. aureus* (MRSA) isolates plays a crucial role in understanding the pathogen's epidemiology, etiology, and antibiotic resistance patterns. This study investigated the genotyping and antibiotic resistance profiles of clinically isolated *S. aureus* strains from different hospitals in Isfahan, Iran. Sixty-three MRSA isolates were analyzed using the disc diffusion method. After DNA extraction, multilocus sequence typing (MLST) was performed using seven housekeeping genes, revealing genetic diversity. Six isolates were selected based on their resistance patterns for MLST. The most frequent isolates were detected from wounds (41.3%), and the lowest frequency was from synovial samples (1.6%). Based on the antibiotic resistance pattern, the highest antibiotic resistance of *S. aureus* isolates was related to tetracycline, ciprofloxacin, and clindamycin at 68.3%, 44.4%, and 44.4%, respectively. In contrast, 96.8% and 95.2% of the isolates were sensitive to nitrofurantoin and linezolid. Among resistant isolates, six sequence types (STs) were identified, including ST74, ST239, ST805, ST531, ST859, and ST5. This study highlights the prevalence, antibiotic resistance, and genetic diversity of MRSA isolates in Isfahan, Iran. The identification of clonal complexes (e.g., CC5, CC8, CC30) suggests clonal spread, emphasizing the importance of surveillance and prevention strategies.

## 1. Introduction

Methicillin-resistant *Staphylococcus aureus* (MRSA) is a major public health concern due to its high prevalence, virulence, and resistance to multiple antibiotics, including methicillin. MRSA infections are associated with significant morbidity, mortality, and healthcare costs, particularly in clinical settings [1–3]. The pathogenicity of *S. aureus* is attributed to various virulence factors, such as adhesins that facilitate tissue adherence and biofilm formation on medical devices, toxins that damage host cells, and enzymes that promote tissue invasion and immune evasion. These factors, combined with its resistance to β-lactam antibiotics conferred by the *mecA* gene, make MRSA a formidable challenge in the treatment of nosocomial infections [4, 5].

The detection and diagnosis of MRSA rely on both phenotypic and genotypic methods. Phenotypic methods, such as antibiotic susceptibility testing and culture-based techniques, are widely used but often limited by their time-consuming nature and lower accuracy. In contrast, genotypic methods, including polymerase chain reaction (PCR), pulsed-field gel electrophoresis (PFGE), and whole-genome sequencing (WGS), offer higher specificity and speed by directly targeting resistance genes and genetic markers [6]. Among these, multilocus sequence typing (MLST) has emerged as a powerful tool for genotyping MRSA isolates. MLST involves sequencing specific housekeeping genes to assign sequence types (STs), enabling detailed epidemiological investigations and the identification of high-risk pandemic clones, such as ST239, which are associated with increased transmissibility and resistance patterns [7]. Despite advancements in diagnostic techniques, the continuous evolution of MRSA strains necessitates ongoing research to improve detection methods and understand their genetic diversity and resistance profiles [8, 9]. This study aims to genotype clinical MRSA isolates from Isfahan, Iran, using the MLST method to investigate their genetic diversity and antibiotic resistance patterns. The findings will contribute to a better understanding of MRSA epidemiology in the region and inform strategies for infection control and treatment.

## 2. Materials and Methods

### 2.1. Study Design and Samples

This cross-sectional study has been conducted on 63 MRSA isolated from different hospitalized patients in Isfahan, Iran, from 2020 to 2021. The study was confirmed by the Shahid Ashrafi Esfahani University, Isfahan, Iran. These samples were collected as part of standard patient care, and no additional samples were required.

Inclusion Criteria: Clinical samples must be confirmed as *S. aureus* through standard microbiological methods. Moreover, samples must be obtained from clinical settings, such as hospitals or healthcare facilities, to ensure relevance to nosocomial infection. Furthermore, isolates that do not exhibit methicillin resistance (i.e., MRSA or MSSA) were excluded and nonclinical or environmental samples were not included in this study.

The primary identification of collected isolates was applied using culture and biochemical tests such as colony morphology, Gram staining, catalase test, coagulase test, mannitol fermentation, and DNase test [8].

### 2.2. Antibiotic Susceptibility Profile

Using cefoxitin disks, MRSA isolates were evaluated for antibiotic susceptibility profiles based on CLSI 2022 guidelines [10]. Selected antibiotic discs were tetracycline, ciprofloxacin, clindamycin, gentamycin, doxycycline, rifampin, cotrimoxazole, linezolid, and nitrofurantoin, respectively. MRSA strains were differentiated from MSSA based on the diameter of the observed halos and strains with a diameter of no growth halo ≤ 21 mm were considered MRSA, and strains with a halo diameter ≥ 22 were considered MSSA [10, 11].

### 2.3. PCR for MLST

Genomic DNA was isolated from fresh colonies using a modified previously established method [18]. The purity and concentration of the extracted DNA were measured using a NanoDrop spectrophotometer (Thermo Scientific, Wilmington, USA) [12]. PCR was performed according to the Pasteur profile from https://www.pubmlst.net website to confirm *S. aureus* genome identification using the reference strain of ATCC 25923 running ABI 3730xl (Country) on 12.5 μL Master Mix (Amplicon, Denmark), 1.4 μL forward and reverse primers (Table 1), 10.5 μL DNA, and 2 μL extracted DNase-free-water. The amplification was carried out over 33 cycles, with the following steps: 2 cycles of 94°C for 5 min; 30 cycles of 94°C for 1 min, 52°C–56°C for 30 s, and 72°C for 2 min; and one cycle of 72°C for 2 min) [13]. Selected internal (housekeeping) genes were *arc* (Carbamate kinase), *aroE* (Shikimate dehydrogenase), *glpF* (Glycerol kinase), *gmk* (Guanylate kinase), *pta* (Phosphate acetyltransferase), *tpi* (Triosephosphate isomerase), and *yqi* (Acetyle coenzyme A acetyltransferase). The purity of DNAs was confirmed by NanoDrop spectrophotometer and agarose gel electrophoresis (sharp single band) [14–16].

PCR products of genes were sequenced using Sanger sequencing by Gene Fanavaran Co. (Tehran, Iran). The sequences were submitted for analysis using the Genetics Analysis 11 software (MEGA 11). The data was analyzed using the PHYLOViZ software and employing the Neighbour-Joining (NJ) method with 1000 bootstrap replicates. The goeBURST algorithm was applied, and the clonal relationships of the strains were analyzed about specific clonal complexes (CCs) present in the databases, based on the identified STs using the software https://www.phyloviz.net/ (PHYLOViZ Online) [17].

## 3. Results

Among 63 MRSA isolates, 41.3%, 34.9%, 11.1%, 7.9%, 3.2%, and 1.6% were isolated from the wound, urine, pulmonary, blood, abscess, and synovial samples. Based on the antibiotic resistance pattern, the highest resistance *S. aureus* isolates were reported against tetracycline, ciprofloxacin, clindamycin, gentamycin, doxycycline, rifampin, and cotrimoxazole as 68.3%, 44.4%, 44.4%, 27%, 23.8%, 22.2%, and 19%, respectively. However, 95.2% and 96.8% of the isolates were sensitive to linezolid and nitrofurantoin, respectively.

**Figure 1:**
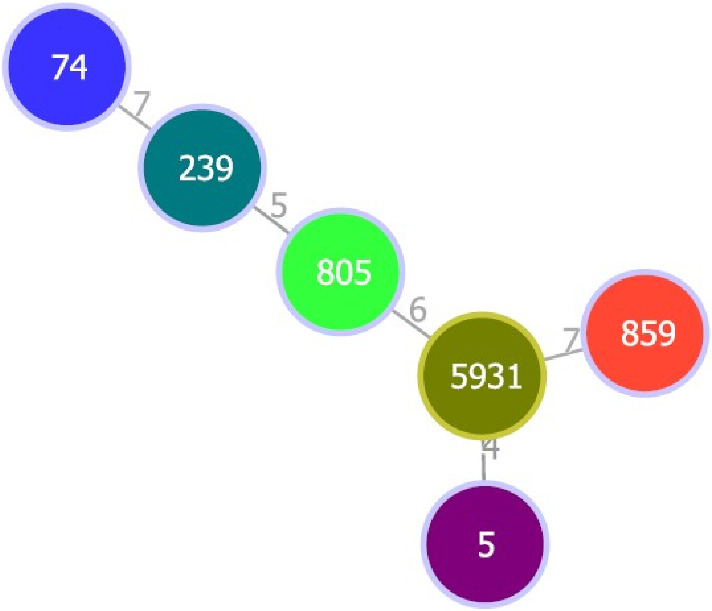
Clonal relationship of STs; one isolate of each ST74, ST239, ST805, ST5931, ST859, and ST5. The numbers (7, 5, 6, 7, 4) represent the number of allelic differences in the seven loci between the STs. For example, the number 4 indicates that two STs differ in 4 housekeeping gene variants. These values illustrate the genetic diversity and relatedness among the MRSA isolates.

**Figure 2:**
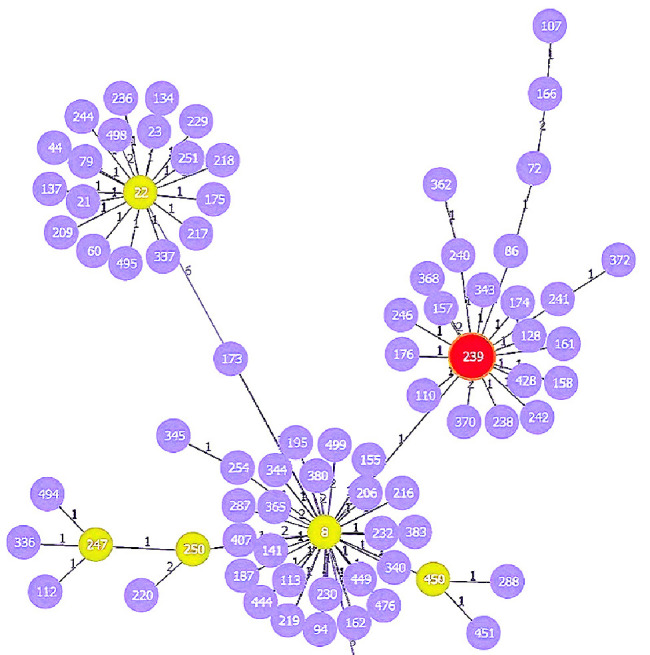
Clonal complex of ST239; ST239 belongs to CC8. 1: Single-locus variant (SLV). 2: Double-locus variant (DLV).

**Figure 3:**
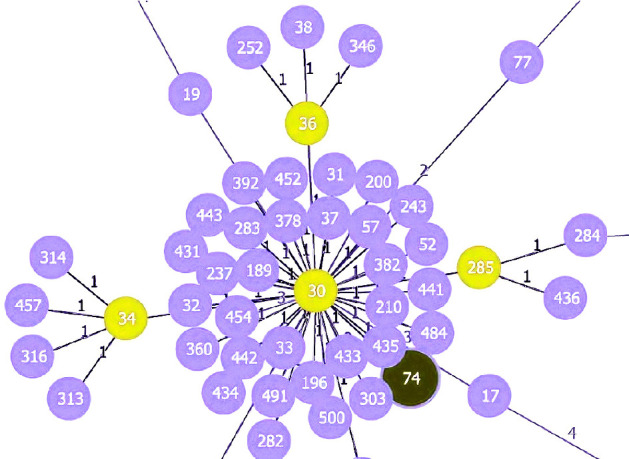
Clonal complex of ST74; ST74 belongs to CC30; 1: Single-locus variant (SLV). 2: Double-locus variant (DLV).

This study selected six isolates for MLST typing based on their resistance patterns. The MLST typing results of the selected isolates, along with the antibiotic resistance pattern, MRSA, and clinical sample type, are presented in Table 2. The STs identified one isolate of each ST74, ST239, ST805, ST5931, ST859, and ST5. The clonal relationship analysis revealed that ST5 and ST5931 belong to the same CC of CC5; ST239 belongs to CC8, ST74 belongs to CC30, and ST859 was identified as a singleton, which does not belong to any CC (Figures 1, 2, 3).

**Figure 4:**
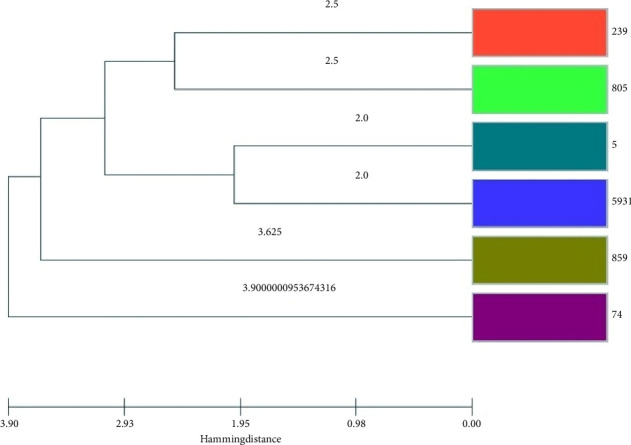
Phylogenic tree of *S*. *aureus*; the colors correspond to various sequence type.

The phylogenetic tree results indicate a high genetic relatedness among the examined isolates, suggesting genetic diversity. Based on the phylogenetic tree, ST239 and ST805 shared genetic relatedness, while ST5 and ST5931 also exhibited genetic relatedness (Figure 4).

## 4. Discussion

### 4.1. Overview of MRSA and Its Clinical Impact

*S. aureus* can produce a wide range of infections that vary in severity, from mild skin infections to life-threatening necrotizing pneumonia. MRSA is of great concern due to its continuous and increasing occurrence of hospital-acquired infections and significant community-acquired infections. Its high prevalence in most regions has significantly increased mortality and treatment costs [13, 18]. Although efforts to control and prevent the spread of this pathogen through patients, health care staff, and environmental screening constitute a significant priority in infection control programs, relying on classification methods as important tools for describing the specific genetic characteristics of isolates can be beneficial for epidemiological investigations to trace the source and identify transmission routes of MRSA [19, 20]. These methods help trace the source and identify transmission routes of MRSA, which is essential for understanding the epidemiology, origin, and spread of MRSA infections [21].

### 4.2. Clinical Sample Analysis

In our study, the most frequently isolated clinical samples were from wounds (41%) and urine (35%). This finding is in line with previous studies, such as Van An et al., who reported 64.6% of MRSA isolates from wound samples [22]. These findings underscore the prevalence of MRSA in wound infections, highlighting its clinical importance [23].

### 4.3. Antibiotic Resistance Profile

The *S. aureus* isolates in this study showed the highest resistance to tetracycline, ciprofloxacin, and clindamycin. A meta-analysis by Dadashi et al. found that the rate of MRSA in Iran ranged from 36% to 50% across four periods over 16 years, with the prevalence observed in blood, wound, and respiratory tract samples [24]. The variation in MRSA prevalence among hospitals can be attributed to differences in sample sizes, sampling methods, culturing techniques, and infection control policies [25]. For example, Goudarzi et al. reported a 71% prevalence of MRSA in Tehran, with the highest resistance to penicillin, kanamycin, gentamicin, and ciprofloxacin, while all MRSA isolates remained sensitive to vancomycin, linezolid, and teicoplanin [26]. On the other hand, Hajimohammadi et al. represented that the highest levels of resistance among MRSA isolates were against erythromycin (80%) and ciprofloxacin (67%) [27]. A similar study found that over 80% of MRSA isolates were resistant to erythromycin, tetracycline, and gentamicin [28]. In a study by Zarkesh et al., the highest resistance was observed against penicillin (100%), followed by erythromycin and ciprofloxacin (92%), while the lowest resistance was observed against chloramphenicol [29]. The widespread use of these antibiotics may contribute to the emergence of resistance, which is further reinforced by the role of clinical isolates in transferring resistance to MRSA strains [30].

### 4.4. Molecular Characterization of MRSA Isolates

In this study, we identified six MRSA genotypes: ST74, ST239, ST805, ST5931, ST859, and ST5. ST239, in particular, is one of the most frequently detected genotypes in *S. aureus* isolates worldwide. Goudarzi et al. reported that ST239, along with ST15, ST182, ST22, ST585, and ST123, was predominant in Iranian hospitals [26].

Additionally, a molecular epidemiology study by Zamani et al. showed similar results, with ST22, ST239, and ST5 being the most common genotypes [31]. Our analysis demonstrated that the prevalent CC circulating in Isfahan's hospitals were CC30, CC1, CC5, and CC8. Notably, ST239, which belongs to CC8, was found in a multidrug-resistant isolate from a wound sample, remaining sensitive only to linezolid.

ST239 is the oldest pandemic clone and is considered one of the most successful MRSA lineages in many regions worldwide. This clone is recognized as the predominant clone in Iranian hospitals, European countries, the United States, and some Asian countries [32, 33]. Studies have shown that most MRSA strains in Korea and Japan are associated with ST5, while ST239 is predominant in countries such as China, Indonesia, Singapore, Sri Lanka, and Vietnam. Faraji et al. reported in their meta-analysis that the ST239-MRSA clone is prevalent among Iranian patients, suggesting that the circulation of this clone within the country may serve as a predictor of treatment failure, increased morbidity, and higher mortality rates among hospitalized individuals [9].

Havaei et al. reported that out of 83 isolated *S. aureus* strains from clinical samples, ST239 was the main epidemic MRSA clone (EMRSA). They also demonstrated that ST859 was only resistant to tetracycline with a negative PVL phenotype [34]. In the present study, we have identified a single isolate with the ST859 genotype from blood samples belonging to the CC88 group. This particular isolate showed resistance exclusively to tetracycline and cefoxitin. Similar to our findings, in another study to determine the characteristics of MRSA strains in Iran, it was shown that the ST859 was resistant to tetracycline and fusidic acid and known as a PVL-negative HA-MRSA clone in Iran, which is consistent with our findings [28]. In a study by Najafi Olya et al., 28 *S. aureus* isolates were sequenced using MLST, and the results showed that the isolates belonged to 21 unique STs and the majority of isolates belonged to CC30 [35]. Our findings genotyped ST74 from CC30 for the first time in clinical samples in Isfahan, Iran.

### 4.5. Clinical Implications and the Importance of Molecular Methods

The emergence of MRSA types with multidrug resistance, some of which carry important virulence genes, can seriously threaten healthcare providers and infection control personnel [36]. Given the high diversity of Staphylococcal strains, the role of the hospital environment in the formation of hospital outbreaks should be considered. Due to the importance of the spread of MRSA strains, the use of molecular methods for epidemiological investigations of these strains appears necessary [37].

### 4.6. Limitations

One of the primary limitations of this study is the relatively small number of isolates chosen for MLST analysis. Although this sample provides valuable initial insights into the genetic diversity of MRSA in Isfahan, it may not comprehensively reflect the broader genetic diversity of MRSA strains circulating within the region.

## 5. Conclusion

This study highlights the prevalence, antibiotic resistance, and genetic diversity of MRSA isolates in Isfahan, Iran. High resistance to tetracycline, ciprofloxacin, and clindamycin underscores the need for rational antibiotic use and infection control measures. The identification of CCs (e.g., CC5, CC8, CC30) suggests clonal spread, emphasizing the importance of surveillance and prevention strategies. Isolates remained highly sensitive to linezolid and nitrofurantoin, offering effective treatment options. These findings call for enhanced antimicrobial stewardship and targeted interventions to combat MRSA's clinical and economic impact, improving patient outcomes and reducing its spread in healthcare settings.

## Figures and Tables

**Table 1:** Primers used for MLST of *S. aureus*.

Genes	Amplicon size (bp)	Sequences	Annealing temperature (°C)
*arc*	F: arc up-5′ TTG ATT CAC CAG CGC GTA TTG TC-3′ R: arc dn-5′ AGG TAT CTG CTT CAA TCA GCG-3′	456 bp	53

*aro*	F: aro up-5′ ATC GGA AAT CCT ATT TCA CAT TC-3′ R: aro dn-5′ GGT GTT GTA TTA ATA ACG ATA TC-3′	456 bp	56

*glp*	F: glp up-5′ CTA GGA ACT GCA ATC TTA ATC C-3′ R: glp dn-5′ TGG TAA AAT CGC ATG TCC AAT TC-3′	465 bp	58

*gmk*	F: gmk up-5′ ATC GTT TTA TCG GGA CCA TC-3′ R: gmk dn-5′ TCA TTA ACT ACA ACG TAA TCG TA-3′	429 bp	52

*pta*	F: pta up-5′ GTT AAA ATC GTA TTA CCT GAA GG-3′ R: pta dn-5′ GAC CCT TTT GTT GAA AAG CTT AA-3′	474 bp	54

*tpi*	F: tpi up-5′ TCG TTC ATT CTG AAC GTC GTG AA-3′ R: tpi dn-5′ TTT GCA CCT TCT AAC AAT TGT AC-3′	402 bp	55

*yqi*	F: yqi up-5′ CAG CAT ACA GGA CAC CTA TTG GC-3′ R: yqi dn-5′ CGT TGA GGA ATC GAT ACT GGA AC-3′	516 bp	58

*Note:* F, forward; R, reverse.

**Table 2:** Distribution of 6 MRSA isolates according to MLST profile, clinical sample, and antibiotic-resistant pattern.

ST	Allelic profile (arc-aro-glp-gmk-pta-tpi-yqi)	CC	Clinical samples	Antibiotic-resistant pattern
239	2-3-1-1-4-4-3	8	Wound	TE-RA-GM-CP-D-CC-SXT-FOX
5	1-4-1-4-12-1-10	5	Wound	CP-CC-FOX
805	3-3-1-1-1-1-1	1	Urine	TE-D-FOX
5931	12-864-1-4-12-1-3	5	Urine	TE-LZ-FOX
859	79-1-14-23-12-4-31	—	Blood	TE-FOX
74	2-2-2-19-6-3-2	30	Blood	GM-CP-CC-SXT-FOX

*Note:* TE, tetracycline; RA, rifampicin; GM, gentamicin; CP, ciprofloxacin; LZ, linezolid; FM, nitrofurantoin; D, doxycycline; SXT, trimethoprim/sulfamethoxazole; FOX, cefoxitin; CC, clindamycin.

## Data Availability

The datasets used and/or analyzed in the current study are available from the corresponding author upon reasonable request.
